# CRISPR/Cas9 Technique for Temperature, Drought, and Salinity Stress Responses

**DOI:** 10.3390/cimb44060182

**Published:** 2022-06-08

**Authors:** Xiaohan Li, Siyan Xu, Martina Bianca Fuhrmann-Aoyagi, Shaoze Yuan, Takeru Iwama, Misaki Kobayashi, Kenji Miura

**Affiliations:** 1 Graduate School of Life and Environmental Sciences, University of Tsukuba, Tsukuba 305-8572, Japan; s2220964@s.tsukuba.ac.jp (X.L.); s2220968@s.tsukuba.ac.jp (S.X.); s2136022@s.tsukuba.ac.jp (M.B.F.-A.); yuanshaoze1994@yahoo.co.jp (S.Y.); s2220921@s.tsukuba.ac.jp (T.I.); s2220938@s.tsukuba.ac.jp (M.K.); 2 Tsukuba-Plant Innovation Research Center, University of Tsukuba, Tsukuba 305-8572, Japan

**Keywords:** abiotic stress, CRISPR/Cas, drought, genome editing, salinity, TALEN, temperature

## Abstract

Global warming and climate change have severely affected plant growth and food production. Therefore, minimizing these effects is required for sustainable crop yields. Understanding the molecular mechanisms in response to abiotic stresses and improving agricultural traits to make crops tolerant to abiotic stresses have been going on unceasingly. To generate desirable varieties of crops, traditional and molecular breeding techniques have been tried, but both approaches are time-consuming. Clustered regularly interspaced short palindromic repeat/Cas9 (CRISPR/Cas9) and transcription activator-like effector nucleases (TALENs) are genome-editing technologies that have recently attracted the attention of plant breeders for genetic modification. These technologies are powerful tools in the basic and applied sciences for understanding gene function, as well as in the field of crop breeding. In this review, we focus on the application of genome-editing systems in plants to understand gene function in response to abiotic stresses and to improve tolerance to abiotic stresses, such as temperature, drought, and salinity stresses.

## 1. Introduction

According to the World Meteorological Organization (WMO), atmospheric greenhouse gas concentrations and the associated accumulated heat have hit record highs. In the last ten years, conflicts, global climate changes, and economic shocks have increased in frequency and intensity. The combined consequences of these threats, exacerbated by the COVID-19 pandemic, have resulted in about a 20% rise in hunger, from 135 million people in 2020 to 161 million by September 2021 [1].

Extreme weather events affect crop yield and food production, which exert inimical effects on the agronomic industry and, thus, increase the demand for agricultural products [2]. Increased concentrations of atmospheric CO_2_ are expected to improve water use efficiency and photosynthetic rates in crops and increased cumulative temperatures may prolong the growing season for crops and reduce growth cycles, particularly in mid-latitudes location [3]. However, it has been strongly proven that the negative impacts of climate change on agricultural and terrestrial food production are more common than the positive ones. Food security is facing large global and regional risks owing to climate change and increasing food demand. Thus, food production is an important aspect of food security [4]. Major abiotic stressors, including drought, extreme temperatures, and salinity, cause extensive losses in agricultural production [5]. It is estimated that adverse environmental stresses reduce crop yield from 50 to 70%. [6]. The process of plant responses to abiotic stresses is complicated due to the interactions between stressors and diverse molecular, biochemical, and physiological aspects [7]. The development of plant tolerance to abiotic stress is considered a promising approach for increasing crop yields. The knowledge and progress of the structural and functional components of crop genomes have been aided by modern genetics and breeding approaches. Advances in genomics and genome editing have broadened the chances of crop improvement [8]. Since modifying a specific genetic locus is an efficient method for improving breeding, genome-editing technology is increasingly being used in plants.

Because of global population growth, food demand has increased, and sustainable agriculture should be promoted to address the issues of climate change. It is necessary to increase crop varieties to cope with abiotic stresses. Traditional selection or breeding methods, such as marker-assisted selection, are ways to produce crop varieties. In addition, genome editing technologies, in particular the clustered regularly interspaced short palindromic repeats (CRISPR)/CRISPR-associated protein 9 (Cas9) system, are biotechnological techniques for precise genetic modification of crops and can increase varieties with a reduction of time consumption for breeding. In this review, we describe the CRISPR/Cas9 system and highlight the use of the technique to produce plant and crop varieties for temperature, drought, and salinity stress responses. Valuable information concerning the tolerance and sensitive to these stresses is described. The information about tolerance to these stresses can be used for future molecular-based breeding approaches to produce these stress-tolerant plants, and the information about sensitivity can also be useful to understand the molecular mechanisms of the responses to temperature, drought, and salinity stresses. We list the information of several kinds of plants, including *Zea mays* (maize), *Oryza sativa* (rice), *Arabidopsis thaliana* (Arabidopsis), *Solanum lycopersicum* (tomato), *Glycine max* (soybean), *Triticum aestivum* (wheat), and *Solanum tuberosum* (potato). The list provides important information about targets for genome editing in breeding new abiotic stress-tolerant varieties or the application of this information to other plants.

## 2. Genome-Editing Technology

Genome editing, a revolutionary and accurate genetic-engineering technology that can modify specific target genes of the organism genome, is increasingly used in many fields including plant science and crop breeding [9]. Genome-editing technology plays a role in the characterization of gene function and crop improvement [10]. There are three main types of genome-editing technology: zinc-finger nucleases (ZFNs), transcription activator-like effector nucleases (TALENs), and the CRISPR/Cas system [11].

**Figure 1:**
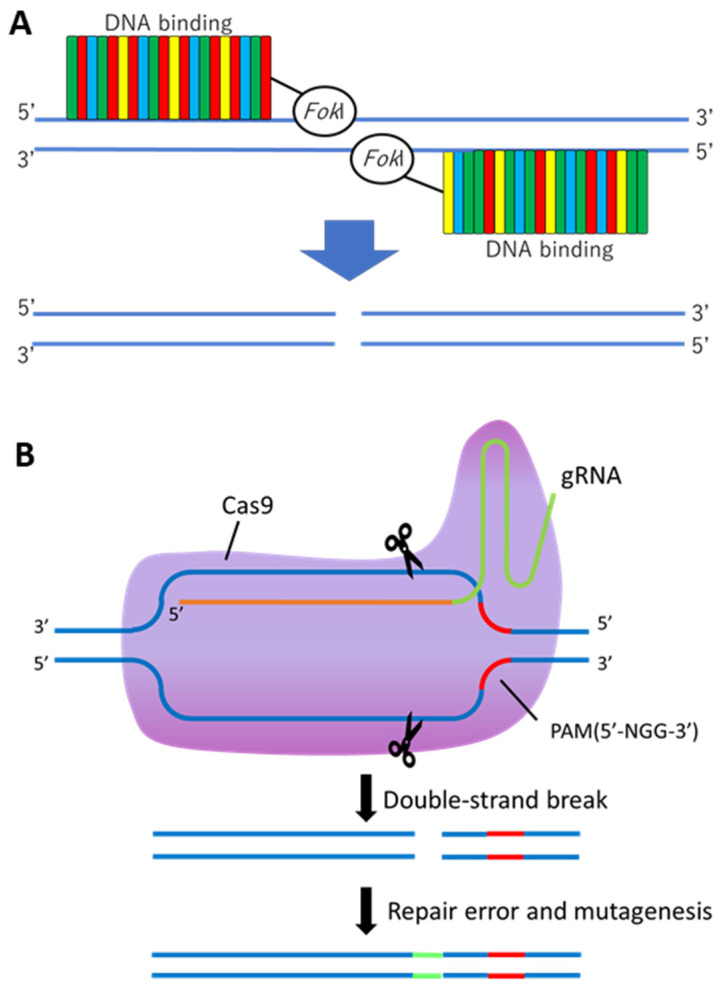
(**A**) A scheme of the TALEN architecture and genome-editing mechanism. Transcription activator-like effector nucleases (TALEN) are chimeric protein that works in pairs composed by a TAL effector DNA-binding domain merged to a nuclease domain from the *Fok*I restriction enzyme. The TALEs have highly conserved and repetitive peptide modules containing up to 34 amino acids (represented by the colored bars). Each TALE repeat specifically recognizes one of the nucleotide bases, and multiple TALE repeats are combined to target a specific DNA sequence and generate a DNA double-strand break by *Fok*I action within the intervening spacer region. (**B**) Overview of CRISPR/Cas9-mediated genome-editing mechanism for non-homologous end joining (NHEJ) repair. Guide RNA (gRNA) is designed to recognize the target sequence located upstream of the protospacer-associated motif (PAM), NGG in the case of CRISPR/Cas9, which serves as a binding signal for Cas9. When nucleotide base pairing occurs (due to the annealing of the target sequence with the protospacer region of the gRNA, represented in the figure by an orange line), the Cas9 enzyme is activated, causing DNA double-strand break. The breaks activate the intracellular repair systems of the cell, which convert breaks into insertion or deletion mutations. The mutations generally cause sequence failures and generate non-functional proteins. CRISPR-associated protein 9 (Cas9); guide RNA (gRNA); protospacer adjacent motif (PAM).

A wide spectrum of genetic alternation has been performed by ZFNs and TALENs via inducing DNA double-strand breaks, which stimulate error-prone non-homologous end joining (NHEJ) or homology-directed repair (HDR) at specific genomic locations [12]. A class of targeting reagents, ZFNs, consists of zinc-finger-based DNA-recognition modules and the DNA cleavage domain of the *Fok*I restriction enzyme [13,14]. Each individual zinc finger recognizes and attaches to a nucleotide triplet and then assembles into groups to bind to particular DNA sequences. Because ZFNs with a high sequence affinity are complex to design and their off-target efficiency is high [15], it took 9 years from the development of ZFNs to the success of the first ZFN-based plant genome editing [16]. The transcriptional activator-like effector (TALE) repeats and the *Fok*I restriction enzyme are fused together to form TALENs (Figure 1A). The central domain of TALE consists of a repeating unit composed of approximately 34 amino acids. The specificity of TALEs depends mainly on repeat variable diresidues (RVDs) and hypervariable amino acids at the 12th and 13th positions. Four different amino acid RVDs (NI, HD, NG/HG, and NN) were used to identify adenine (A), cytosine (C), thymine (T), and guanine (G)/adenine (A), respectively. The DNA identifiers provide the intimate connection between the network of amino acid repeats and the nucleotide sequence of the genome; hence, TALENs can be engineered with desired sequence properties [17].

Currently, a popular and powerful gene-editing tool is the CRISPR/Cas9 system, because of its simplicity and usability. Cas enzymes are divided into two classes (Classes I and II) depending on the architecture of interference effector modules and six major types (types I–VI); 33 subtypes exist based on the signature *cas* gene and distinct mechanism of targeting [18]. As part of the bacterial adaptive immune system, CRISPR sequences can recognize exogenous viral DNA and directly cleave Cas proteins [19]. Among these subtypes, the CRISPR/Cas9 system has been extensively studied. The Cas9 protein and synthetic single-guide RNA (sgRNA), which is generated by fusing CRISPR-RNA (crRNA) and transactivation RNA (tracrRNA), are the primary components of the CRISPR/Cas9 system. The site-specific DNA double-strand breaks (DSBs) are introduced by sgRNA-guided Cas9 protein, thus triggering DNA repair mechanisms (Figure 1B). The target sequence located upstream of the protospacer-associated motif (PAM), NGG, in the case of CRISPR/Cas9, was designed. The system has been widely used in plants for characterization of gene function and precision plant breeding [20,21,22].

Although the CRISPR/Cas9 system is effective, other Cas proteins have been identified and applied in genome-editing technology. Cas12a/Cpf1 is the most notable Cas protein in addition to Cas9. It recognizes different PAM sequences and produces sticky ends, rather than blunt ends, which facilitates HDR repair to produce more precise editing [23]. Among the Cas enzyme subtypes, some have been applied to plants, such as Cas3 [24], Cas10 [24], Cas12 [23], Cas12b [25], Cas13a [26,27], and CRISPR/CasΦ [28]. These different enzymes may increase the range of options available for different plant species. SpCas9 derived from *Streptococcus pyogenes* has been widely used. Compared to SpCas9, SaCas9 derived from *Staphylococcus aureus* is smaller and more conducive to intracellular delivery [29]. In addition, researchers have identified many Cas proteins targeting different PAM sequences, such as StCas9 (from *Streptococcus thermophilus*), NmCas9 (from *Neisseria meningitidis*), FnCpf1 (from *Francisella novicida*), and so on [30].

Base-editing technology has become another option for effective and accurate genome editing. The base editor enables precise substitution of individual nucleotides. Fusion of dead (dCas9) or nickase Cas9 (nCas9) with cytidine base editing (CBE) and adenine base editing (ABE) can achieve base editing from C–G to T–A and from A–T to G–C, respectively [31,32]. Because single-nucleotide mutations are important sources of superior crop traits [33,34], base editors can improve crop quality.

In polyploids, many genes consist of several copies, named homologs, which have the same role in determining certain plant features. These homologs must be altered concurrently in order to produce recessive mutants. Furthermore, members of the gene family usually share comparable structures and activities, contributing to genetic robustness. Therefore, it will be more efficient to mutate two or more paralogs to show a phenotypic effect rather than knocking out one gene. The multiplex genome-editing technique is a worthwhile technique to genetically alter several gene functions [35]. This technique has demonstrated its great superiority in editing multiple target sites simultaneously. Recent research using multiplex genome editing in plants has been predominantly carried out by the CRISPR/Cas system, because an sgRNA can target several homologous alleles. If a conserved sequence exists in several genes, an sgRNA can target them all or multiple gRNAs can also be employed to target genes that contain no conserved sequence [36].

Prime editing is an emerging genome-editing technology to achieve precise editing and consists of two parts: one is the effector protein formed by the fusion of nCas9 and the engineered reverse transcriptase (RT); the other is the prime editing guide RNA (pegRNA) including sgRNA, primer binding site (PBS), and RT template with edit. A pegRNA is a special gRNA that guides the edited protein to the target site and contains the edited template sequence. The Cas9-RT fusion protein precisely cuts a DNA strand under the guidance of pegRNA and then synthesizes DNA with the correct sequence according to the template. The DNA repair machinery in the cell automatically integrates this newly synthesized sequence into the genome [37]. Compared with the CRISPR/Cas9 system, the restriction of PAM sequences on prime editing is greatly reduced. For prime editing, the distance from the PAM to the editing site can exceed 30 base pairs (bp). Prime editing can achieve all 12 base substitutions, while the base editor can only perform four types of substitutions [38]. Even though CRISPR/Cas9-mediated genome editing can be used for HDR after double-strand breaks, the efficiency of HDR production in plants is very low. In contrast, the advent of pregRNAs has greatly improved editing efficiency. Because 33 of 62 transgenic lines harbor the substation of serine 621 to isoleucine (S621I) in the *ZmALS1* and/or *ZmALS2* genes, the editing efficiency of prime editing in maize is as high as 53.2% (33/62) [39]. As an emerging genome-editing method, prime editing still needs more exploration. However, its own characteristics of high efficiency, precision, and site-directed mutation make it have infinite prospects and wide applications in future gene-editing projects.

## 3. Abiotic Stress Responses Revealed by Genome Editing

One of the innovative techniques, the CRISPR/Cas9 system precisely introduces mutation(s) in the target gene(s), resulting in enhancement of tolerance to abiotic stresses or the discovery of the mechanism of signaling. Table 1 summarizes the application of CRISPR/Cas9 to identify the genes for response to abiotic stresses, such as temperature, drought, and salinity stresses.

### 3.1. Temperature Stress Responses

Heat and cold stresses can have dramatic impacts on agriculture the and ecology of plants. Leaf photosynthesis, biomass accumulation, and grain yield are affected by cold stress [79].

Proline-rich proteins function in a variety of physiological and biochemical processes for plant growth and stress responses. The knockout of *OsPRP1*, which encodes a proline-rich protein (PRP) in rice generated by CRISPR/Cas9, enhances cold sensitivity in rice [40]. The CRISPR/Cas9-mediated knockout mutant of *OsMYB30*, which has been characterized as a cold-responsive R2R3-type MYB gene in rice, exhibits better cold tolerance than wild-type rice [41].

The three tandemly arranged *CCAAT-binding factor* (*CBF*) genes—*CBF1*, *CBF2*, and *CBF3*—have been verified to be involved in cold acclimation using the CRISPR/Cas9-mediated Arabidopsis *Atcbf* single, double, and triple mutants. Compared with wild-type Arabidopsis plants, cold-acclimated *Atcbf* triple mutants exhibit extremely sensitive phenotype to freezing temperature. Expression of *CBF* genes is rapidly increased when plants are exposed to cold temperatures. The CBF proteins enhances the transcription of downstream cold-responsive (COR) genes to increase the freezing tolerance of plants [42]. The freezing sensitivity ranking is triple *cbf* mutant > *cbf1 cbf3* double mutant > *cbf3* mutant [43]. Among the tomato *CBF* gene family, *SlCBF1* is the only cold-inducible gene [80]. Both salicylic acid (SA)- and hydrogen peroxide (H_2_O_2_)− induced cold tolerance are achieved by increased *SlCBF1* expression in tomato [44]. The *Slcbf1* mutants generated by the CRISPR/Cas9 system exhibited greater electrolyte leakage and malondialdehyde (MDA) levels than wild-type plants, indicating that knockout of *SlCBF1* can increase cold-stress-induced membrane damage [45].

Many of the *cis*-regulatory elements in the rice *OsAnn5* promoter region are common promoter elements. Some elements are unique to *OsAnn5*, including the dehydration-responsive element (DRE) core (a *cis*-acting element involved in CBF-mediated cold responsiveness) and MYB recognition sites. In the region between the start codon ATG and −2082 bp of *OsAnn5*, plant hormone-regulatory elements, as well as light-responsive elements are included. This implies that several transcription factors regulate *OsAnn5* expression in rice. Compared with wild-type rice, CRISPR/Cas9-mediated elimination of *OsAnn5* function significantly increases survival rates at the seedling stage under cold stress in rice, demonstrating that *OsAnn5* is a positive factor that regulates cold stress tolerance at the seedling stage [46]. Plant annexins are calcium-ion (Ca^2+^)-dependent phospholipid-binding proteins that are implicated in the regulation of plant development and protection from environmental stresses. *OsAnn3*-knockoout mutants generated by CRISPR/Cas9 exhibit an increase in relative electrical conductivity (REC) and a decrease in survival rate compared with wild-type plants, which proves that *OsAnn3* plays a role in cold tolerance in rice [47].

Heat stress adversely affects biological activities, such as chlorophyll content, turgor, photosynthetic rate, carbon assimilation, acquisition of nutrient, cellular metabolism, and gaseous exchange at the leaf surface. Together, these undesirable influences cause a reduction in crop yield [81,82]. The deletion mutants of *heat-stress sensitive albino 1 (OsHSA1)* generated by CRISPR/Cas9 exhibit greater sensitivity to heat than wild-type rice plants [48]. *OsHSA1* encodes fructokinase-like protein2 and functions in chloroplast development and chloroplast protection under heat stress during the early and late stages in rice, respectively. Tomato *Slcpk28* (calcium-dependent protein kinase28) mutants produced by CRISPR/Cas9 exhibit increased heat-stress-induced accumulation of reactive oxygen species (ROS) and protein oxidation level and decreased ascorbate peroxidase (APX) activity and other antioxidant enzymes, such as catalase and glutathione reductase, leading to heat sensitivity [49]. CRISPR/Cas9-mediated knockout of *SlMAPK3* exhibited better heat tolerance by reducing ROS accumulation and upregulating the expression of genes encoding heat stress transcription factors (HSFs) and heat shock proteins (HSPs) [50]. The tomato *BRASSINAZOLE RESISTANT 1 (SlBZR1)* gene acts as a critical regulator of the brassinosteroid (BR) response. BRs are a group of steroid plant hormones that can enhance tolerance to a range of abiotic stressors [83]. Heat-stress-induced damage was exacerbated in the *bzr1* mutants, and BR-induced heat stress tolerance was lost through *RESPIRATORY BURST OXIDASE HOMOLOG1 (RBOH1)*-dependent ROS signaling, which is regulated by FERONIA (FER) homologs [51]. Under heat stress conditions, knockout of *OsNAC006* using the CRISPR/Cas9 system displayed an obvious increase in superoxide radical (O_2^−^_) and H_2_O_2_ levels, as well as a decrease in chlorophyll content and the activities of antioxidant enzymes, demonstrating that *Osnac006* may function in heat tolerance by reducing the antioxidant response, which is triggered in response to oxidative stress and mediates photosynthesis [52]. Although several reports provide molecular-based modification, which confers plant tolerance to temperature stresses, more research to identify the functional mechanism is required. To address the global warming issue, heat stress tolerance varieties and identification of the target to increase heat tolerance are necessary. One of the targets could be *PHTOCHROME* (PHY). PHYB has been identified as a thermosensor [84,85], and the *phy* mutant exhibited high tolerance to heat stress in Arabidopsis [86] and tomato [87]. This mutant information is important for the definition of targets of genome editing.

Heat stress is commonly characterized as a temperature rise that exceeds a threshold level for an extended period of time, causing irreversible damage to plant growth and development. Generally, when the ambient temperature is 10–15 °C higher than the optimum range of temperature for crop cultivation, such conditions are defined as heat shock or heat stress [88]. High temperatures can cause high initial rate respiration in pollen, which leads to the exhaustion of endogenous respiratory substrates, subsequent aging, and a loss of mitochondrial activity, resulting in the abortion of the pollen and reduction of the fruit set [89,90]. Parthenocarpy, the development of fruit in the absence of pollination or fertilization, is considered a valuable goal during seedless fruit development because of its fertilization-independence, consumers’ preference for seedless over seeded fruits, and higher fruit quality [91,92]. After screening an ethyl-methanesulfonate (EMS)-mutagenized tomato population for yield under heat stress, a mutant capable of generating high-quality seedless (parthenocarpic) fruit was isolated. Following next-generation sequencing, marker-assisted mapping and CRISPR/Cas9 gene knockout research discovered that the parthenocarpic phenotype was caused by a mutation in the tomato *SlAGAMOUS-LIKE 6 (SlAGL6)* gene encoding MADS-box. Mutations in *SlAGL6* increase tomato yield under heat stress. The tomato *agl6* mutants exhibited facultative parthenocarpy without any pleiotropic effect and developed seedless fruits, which were comparable in both weight and shape to wild-type seeded fruits [53]. Aux/IAA9 (IAA9) is involved in tomato fruit development and represses fruit initiation without fertilization. CRISPR/Cas9-mediated *IAA9 (SlIAA9)* mutant plants exhibited simple leaves instead of wild-type compound leaves, and fruit development of the *Sliaa9* mutant was stimulated before fertilization, leading to parthenocarpy. These mutations are heritable in subsequent generations [54]. DELLA is a negative regulator of gibberellin signaling. Loss-of-function mutations in *SlDELLA,* also called *PROCERA*, exhibit parthenocarpy in tomatoes [93]. The base-edited *Sldella* mutant with the Target-AID system exhibited high gibberellin sensitivity and a parthenocarpic phenotype [55]. These results suggest that genome-editing techniques enhance parthenocarpy in tomatoes. Parthenocarpy is an important trait to increase the yield of tomatoes in summertime without the application of auxin. Because tomatoes are a member of Solanaceae, it is possible to knockout the genes by the genome-editing technique in other Solanaceae, such as peppers and eggplants.

### 3.2. Drought Stress Responses

Drought is one of the abiotic stresses on plants that seriously reduces crop productivity. The tomato *SlLBD40* gene, which belongs to subfamily II of the LATERAL ORGAN BOUNDARIES DOMAIN (LBD) family, is highly expressed in roots and fruits. The average water loss rate of wild-type tomato plants was significantly higher than *Sllbd40* knockout mutants generated using CRISPR/Cas9 [56]. Auxin response factors (ARFs) are key proteins for various physiological processes, such as leaf expansion, lateral root development, and fruit development, in plants. Knocking out *SlARF4* using CRISPR/Cas9 led to lower water loss than in wild-type tomato plants. The leaves of *Slarf4* plants were able to stand upright again after 24 h of re-watering when they were wilted, but the wild-type plants were not [57]. AITRs are a family of novel transcription factors that regulate plant responses to abscisic acid (ABA) and abiotic stress in Arabidopsis. Single, double, and triple *aitr* mutants exhibited increased drought tolerance. Arabidopsis *aitr* mutants with all six *AITR* genes being knocked out by CRISPR/Cas9 exhibited enhanced drought and salt tolerance and reduced sensitivity to ABA, but the plant growth and development of the sextuple mutants were not affected [3]. CRISPR/Cas9-mediated mutation in the open stomata 2 (*OST2*) gene, which encodes a plasma membrane H^+^ ATPase responsible for stomatal response [94], exhibited a high degree of stomatal closure with a low level of water loss, leading to enhanced drought tolerance in Arabidopsis [59]. Abscisic-acid (ABA)-responsive element binding protein 1/ABRE binding factor (AREB1/ABF2) is an important positive regulator for drought stress response. To activate the endogenous promoter of *AREB1*, the CRISPR/dCas9^HAT^ system was employed. Arabidopsis histone acetyltransferase (HAT) enhances chromatin relaxation and promotes gene expression. HAT fused with dCas9 increased the promoter activity, and the expression levels of *AREB1* were considerably upregulated by 2-fold. The plants generated in this study exhibited better survival rates after drought stress [60]. Maize *ARGOS8* negatively regulates ethylene response. Translocation of the *GOS2* promoter to the *ARGOS8* locus using CRISPR/Cas9 and *ARGOS8* variants resulted in elevated levels of *ARGOS8* transcripts. These variants were compared with a wild-type hybrid under drought stress conditions, and their yields did not decrease significantly. These results indicate that *ARGOS8* variants improve maize grain yield under field drought stress conditions [61]. OsERA1 acts as a negative regulator of responses to drought stress in rice. *Osera1* mutant seedlings, which were created using CRISPR/Cas9, were subjected to water-deficit stress. The relative stomatal conductance rates of the mutant plants were significantly lower than those of the wild-type plants. The *Osera1* mutant exhibits increased sensitivity to ABA [62]. Leaf morphology affects plant tolerance to drought, and genes for the curled leaf phenotype by controlling the quantity, size, and arrangement of bulliform cells (BCs) were influenced. The rolled leaf phenotype was observed by the CRISPR/Cas9-based mutagenesis of *Semi-rolled leaf1,2 (SRL1* and *SRL2)* genes. Mutant plants had lower MDA levels and higher content of abscisic acid (ABA) than wild-type plants under drought stress. This indicates that the mutant plants are more drought-tolerant than the wild type [63].

In contrast, some genes have been edited to make plants drought sensitive. These mutants are also useful to understand more precisely the mechanism of drought stress responses. A master regulator, NPR1, is involved in plant defense responses to pathogens. Interestingly, tomato *Slnpr1* mutants created by CRISPR/Cas9 exhibited reduced drought tolerance, and the survival rate of *Slnpr1* mutants was significantly lower than that of wild-type plants under drought conditions [64]. Mitogen-activated protein kinases (MAPKs) are key signaling molecules. Compared with wild-type plants, *slmapk3* mutants created using the CRISPR/Cas9 system exhibited more severe wilting and membrane damage under drought stress in tomato [65]. The MYB-related gene *GmMYB118* improved tolerance to drought stress by reducing the content of ROS and MDA. Using CRISPR/Cas9 to knock out *GmMYB118,* mutant plants exhibited reduced drought tolerance [66]. *OsPUB67*, which encodes a U-box E3 ubiquitin ligase in rice, was drastically induced by drought stresses. A drought-sensitive phenotype was observed in the CRISPR/Cas9 knockout plants. At the seedling stage, the drought stress response of the overexpressing (OE) lines and mutant lines was investigated. The survival rates of the OE lines were significantly greater after re-watering than those of the wild type and mutants. These results suggest that *OsPUB67* has a positive role in drought stress tolerance [67]. Osmotic stress-/ABA-activated protein kinase 2 (SAPK2) is the primary mediator of ABA signaling. Loss-of-function mutants were produced using the CRISPR/Cas9 system. Compared with the wild type, *sapk2* mutants are mostly insensitive to ABA, with relatively low survival rates under drought stress [68].

As indicated, several mutations generated by CRISPR/Cas9 enhanced drought tolerance. It is possible that stacking of mutations by crossing or by multiplexing will show additive effects, enabling the production of plants highly tolerant to drought stress.

### 3.3. Salinity Stress Responses

Salinity is a severe threat to crop yield, because most agricultural plants cannot survive in high-salt environments [95]. Research on salinity tolerance is becoming increasingly important, and enhancement of salt tolerance has become an important breeding goal. The *OsRR22* gene, which encodes a 696-amino-acid B-type response regulator transcription factor, is involved in both cytokinin signal transduction and metabolism. The Cas9-mediated *OsRR22* mutant lines grew better than the wild type under a 0.75% sodium chloride (NaCl) nutrition solution [69]. OsVDE, a lipocalin-like protein in chloroplasts, facilitates ABA biosynthesis and negatively regulates salt-stress tolerance in rice seedlings. The *Osvde* mutant generated by the CRISPR/Cas9 system exhibited greater stomatal closure and higher ABA content than the wild type, resulting in the reduction of water loss from the transpiration process [70]. The drought and salt tolerance (*DST*) gene was selected for CRISPR/Cas9-mediated genome editing in indica rice cv. MTU1010, and different mutant alleles of the *DST* gene were successfully generated. *dst^Δ184–305^* were selected for phenotypic analysis. Under osmotic and salt stress, > 65% of *dst^Δ184–305^* mutant seedlings survived. Furthermore, *dst^Δ184–305^* mutants exhibited higher chlorophyll retention [71]. A total of 158 NAC transcription factors have been identified in rice that are involved in multiple abiotic stresses. To determine the particular role of *OsNAC041* in abiotic stress response, targeted mutagenesis of the *OsNAC041* locus was performed using the CRISPR/Cas9 system. Seed germination and subsequent growth of the *Osnac041* mutants were suppressed at 7 days when treated with 150 mM NaCl, relative to the wild type. Furthermore, shoots of the *Osnac41* mutant seedlings were shorter than those of the wild-type seedlings under salt stress, demonstrating that the mutants were sensitive to salt stress [72].

Hybrid proline-rich proteins (HyPRPs), a subclass of putative plant cell wall glycoproteins, have been shown to be negative regulators of tomato multi-stress responses. Engineering the *SlHyPRP1* gene by precisely removing its PRD, 8CM domain, or both resulted in greater survival rates than the wild type in medium containing 150 mM NaCl [73]. Auxin response factors (ARFs) also have important roles in regulating the expression of auxin response genes in tomatoes. Using CRISPR/Cas9 to obtain *SlARF4* mutants under NaCl exposure, the loss of shoot fresh weight in the mutants was half that of the wild type [74].

AITRs are a family of transcription factors that regulate plant responses to abiotic stresses. In Arabidopsis, there are six genes encoding AITRs. Knockout of any one of these genes using CRISP/Cas9 showed enhanced tolerance to salt treatment, indicating that the entire family of AITR genes in Arabidopsis leads to enhanced salinity tolerance [57]. The *ACQOS* locus is a cluster comprising four nucleotide-binding leucine-rich repeats (NLRs) encoding a toll-interleukin1 receptor-nucleotide-binding leucine-rich repeat class protein. Wild-type Arabidopsis *ACQOS* alleles are salt sensitive. A knockout line of the *ACQOS* allele has been established using the CRISPR/Cas9 system in Arabidopsis. Chlorophyll measurements suggested that *ACQOS* silencing significantly affected salt stress tolerance, because the chlorophyll content was significantly reduced in mutants compared to the wild type [75].

Bread wheat (*Triticum aestivum* L., BBAADD) is a typical allohexaploid species with higher salt tolerance than its tetraploid wheat progenitor (BBAA) [76]. The *TaHAG1* overexpressed *(TaHAG1-OE)* plants were subjected to salt stress together with wild-type plants, and the *TaHAG1-OE* lines exhibited a less severe phenotype. To further verify the function of *TaHAG1* in salt tolerance, *Tahag1* mutants were produced by the CRISPR/Cas9 system. Under salt stress, the wild-type and mutant plants exhibited obvious physiological differences, including a decrease in root length and fresh weight, an increase in chlorotic leaves, and greater Na^+^ content in the mutants. This indicates that TaHAG1 acts as a crucial regulator in strengthening the salt tolerance of hexaploid wheat [76].

Coilin is the major structural protein that controls the formation, composition, and activity of subnuclear Cajal bodies. A fragment of the potato coilin gene encoding the CTD was edited using CRISPR/Cas9 technology. Under salt-stress conditions, the WT exhibited accelerated yellowing, leaf fall, and more severe inhibition of root development, indicating that potato coilin is involved in the plant defense response to salinity [77].

Soybean growth and yield are largely affected by abiotic stresses such as drought, salinity, and extreme temperatures. *AITRs* are ABA-induced transcriptional repressors. Transgene-free *Gmaitr* mutants were generated using CRISPR/Cas9 genome editing to target *GmAITR* genes. Under salt treatment, *Gmaitr* mutant seeds showed a higher germination rate than the wild type, and the *Gmaitr* mutant plants were morphologically similar to the wild-type plants in the normal soil field, but grew better in the saline soil field [78]. Interestingly, CRISPR/Cas9-mediated mutation in the *AITR* gene enhanced salinity stress tolerance in Arabidopsis and soybean. Furthermore, the *aitr* mutant also exhibited drought tolerance in Arabidopsis [58]. It is plausible that the *AITR* gene is one of targets for CRISPR/Cas9-mediated mutagenesis to increase tolerance to salinity stress in several plant species.

## 4. Perspectives

A growing world population and increasingly frequent climate extremes threaten human food security [96]. However, humans have limited means to increase food production, and traditional mutation breeding techniques rely on unpredictable genomic mutations and labor-intensive screening, resulting in inefficiencies [97]. There is an urgent need for rapid, targeted breeding methods to accelerate plant improvement. Genome editing is a useful technology in crop breeding [9]. In contrast to earlier genetic engineering techniques, which involve the random insertion of a foreign gene into the plant genome, genome-editing technology can manipulate the genome of organisms more precisely [98]. Thus, genome-editing technology has facilitated the acquisition of plants with characteristics of interest. However, several challenges remain to be overcome.

### 4.1. Cost and Regulation Aspects

One of the main limitations of genome-editing techniques is the cost of producing and using zinc fingers and TALEN. Since the discovery and rise of CRISPR/Cas9, the cost, time, and work required to obtain genome-edited plants have significantly decreased [19,99,100]. Gene-edited products have been available on the market since 2012, when an article about CRISPR/Cas9 was published [101]. The first available product was Calyno, a high-oleic soybean oil produced by Calyxt in the U.S. [102]. In addition to genome-edited soybean, herbicide-tolerant canola by Cibus in Canada and γ-aminobutyric-acid (GABA)-rich tomato have been commercially provided by Sanatech Seed in Japan [103]. Furthermore, genome-edited fishes, such as “Madai” red sea bream with an improved feed utilization efficiency and “22-seiki fugu” tiger puffer for faster growth, have reached consumers.

In addition, to facilitate access to gene-editing technology, unlike techniques for obtaining genetically modified organisms (GMOs), genome-editing techniques do not require the insertion of exogenous DNA, making them unsuitable for the regulatory regimes of GMOs [104]. Despite this, the EU and other countries such as New Zealand have adapted their legislation and consider genome-edited plants as GMOs, stigmatizing their commercialization and acceptance [105]. Research has already shown that the public is more receptive to the consumption of CRISPR-derived products than of GM-derived products [106]. Recently, the European Commission started to reconsider the GMO rules for gene-edited crops. It is possible that genome-edited foods will be widely available on the market.

### 4.2. Genome-Editing Tools Delivery Limitations

*Agrobacterium*-based tissue culture methods are often used to deliver CRISPR/Cas9 expression cassettes in plants. However, there are still limitations related to tissue culture and plant regeneration. Tissue culture is time consuming and laborious. Some innovative genome-editing delivery methods have recently emerged, such as viral delivery, nanoparticle (NP) delivery, and meristem induction (Table 2). The virus is an efficient DNA-delivery tool that can deliver sgRNAs; however, it is difficult to use viruses to deliver complete CRISPR/Cas9 expression cassettes because of the limited loading capacity of viral capsid proteins [107]. Rhabdovirus and potato virus X can deliver the complete CRISPR/Cas9 expression cassette and can be successfully applied for genome editing in *Nicotiana benthamiana* [108,109]. NPs can deliver CRISPR/Cas9 expression cassettes to plant protoplasts [110], and cationic lipid NP-based genome editing has been achieved in citrus [111]. However, the size of the NPs is still large, making it difficult to deliver the complete CRISPR/Cas9 expression cassette through the cell wall. The meristem-induction method is a genome-editing method that does not rely on tissue culture. This method introduces both plant growth regulators and CRISPR/Cas9 expression cassettes into plant leaves, thereby inducing meristematic tissues in the leaves. This method achieves relatively high genome-editing efficiency in *Nicotiana benthamiana* [112]. In addition, transient expression techniques have been used to improve base editing in various plants [113,114,115,116,117]. Exogenous DNA may not integrate into the plant genome, which facilitates the rapid cultivation of transgene-free plants. The in planta particle bombardment (iPB) method is another method to deliver DNA or CRISPR/Cas9-ribonucleoprotein to shoot apical meristem cells [118]. In wheat, 5.2% of plants carried mutant alleles, and mutations in three of these were also inherited in the following generation. Because *Cas9* and sgRNA were not detected, the transient expression of CRISPR/Cas9 introduced mutations [119]. This technique has also been successfully applied to other species such as sorghum [120], japonica rice [121], onion, and *Nicotiana benthamiana* [122].

To increase the efficiency and accuracy of Cas9, many strategies for and modifications to the enzyme have been developed. The intronized *Cas9* gene has been studied and exhibited an increase in the mutation rate from 70% to 100% in comparison with the non-intronized *Cas9* gene in *Arabidopsis thaliana*, *Nicotiana benthamiana*, and *Cartharanthus roseus* [123]. Because the ideal growth condition for *Streptococcus pyrogenes* is 40 °C, the efficiency of on-target mutations after incubation cycles at 37 °C was increased in *Arabidopsis thaliana* and citrus [124]. However, this is not an ideal temperature for the growth of many plants. The Cas enzyme, which works at lower temperatures and even responds to light, has been developed [125].

These innovative DNA delivery methods for increased efficiency can overcome the limitations of *Agrobacterium*-based genome editing and accelerate the process of gene editing-based plant breeding.

### 4.3. Broadening Gene Targets and Crops

Another challenge is found in editing the genome of polyploid species, since many studies and products obtained have focused on diploid species or model plants. In polyploid species, mutations that cause a loss of function are usually inefficient because of genetic redundancy [126,127]. In the case of CRISPR/Cas9, multiple gRNAs can be used, and mutations in different alleles can be introduced. In addition to wheat, this technique can be successfully applied to cotton [128], strawberries [129], and potatoes [130].

## 5. Conclusions

ZFNs, TALENs, and CRISPR/Cas systems have been used for different purposes in modern agriculture, such as obtaining plants with tolerance to abiotic stresses, such as temperature, drought, and salinity stresses. With the emergence of CRISPR/Cas, these processes have become more rapid and accurate. In recent years, many advances have been made in genome-editing technologies, such as the modification of known structures or the discovery of new enzymes or methodologies. With the advancement of biotechnology, new tools can be explored and improved to overcome barriers and the surprising progress in the genome-editing system in plants.

## Figures and Tables

**Table 1:** Application of CRISPR/Cas9-mediated genome editing to elucidate genes involved in response to temperature, drought, and salinity stresses.

Plant Species	Target Genes	Gene Function	Phenotype	Mode of Application	Ref.
Rice	*OsPRP1*	Proline-rich protein	Cold sensitive	Mutants exhibited sensitive phenotype after treatment at 6 °C for 3 days.	[40]
Rice	*OsMYB30*	Transcription factor	Cold tolerance, increased panicle length, enlarged grain size	Mutants exhibited tolerance phenotype after treatment at 4 °C for 5–10 days.	[41]
Arabidopsis	*CBFs*	Transcription factor for abiotic stress responses	Extremely sensitive to freezing	Mutants exhibited sensitive phenotype after treatment at 4 °C and freezing for 7 days and freezing-sensitive phenotype after treatment at −7 °C for 1 h.	[42]
				Mutants exhibited sensitive phenotype after treatment at 4 °C for 50 days and freezing sensitive after treatment at −9 °C for 1 h and −10 °C for 1 h.	[43]
Tomato	*SlCBF1*	Transcription factor for abiotic stress responses	More severe chilling injury symptoms	Mutants exhibited sensitive phenotype after treatment at 4 °C for 7 days.	[44,45]
Rice	*OsAnn5*	Annexin	Cold tolerance	Mutants exhibited tolerance phenotype after treatment at 4–6 °C for 3 days.	[46]
Rice	*OsAnn3*	Annexin	Cold tolerance	Mutants exhibited tolerance phenotype after treatment at 4–6 °C for 3 days.	[47]
Rice	*OsHSA1*	Fructokinase-like protein 2	Heat sensitive	Mutants exhibited tolerance phenotype after treatment at 32 °C for 60 days.	[48]
Tomato	*SlCPK28*	Protein kinase, Ca^2+^ sensing	Heat sensitive, accumulation of ROS	Mutants exhibited sensitive phenotype and higher H_2_O_2_ content after treatment at 45 °C for 12 h.	[49]
Tomato	*SlMAPK3*	MAP kinase upregulating HSPs’/HSFs’ genes’ expression	Heat tolerance, reduction of ROS accumulation	Mutants exhibited tolerance phenotype and lower H_2_O_2_ and O_2_^•−^ contents after treatment at 42 °C for 1 day.	[50]
Tomato	*SlBZR1*	Transcription factor for brassinosteroid response	Heat tolerance	Mutants exhibited tolerance phenotype after treatment at 42 °C/38 °C (day/night) for 1 day.	[51]
Rice	*OsNAC006*	NAC transcription factor	Heat sensitive	Mutants exhibited sensitive phenotype after treatment at 42 °C for 4 days.	[52]
Tomato	*SlAGL6*	MADS-box	Parthenocarpy, tomato fruit under heat stress	Mutants exhibited facultative parthenocarpy phenotype after treatment under natural heat stress for 67 days.	[53]
Tomato	*SlIAA9*	Transcriptional regulator for auxin response	Parthenocarpy	Mutants exhibited parthenocarpy phenotype.	[54]
Tomato	*SlIAA9*	Transcriptional regulator for auxin response	Parthenocarpy	Mutants exhibited parthenocarpy phenotype.	[55]
Tomato	*SlLBD40*	Plant-specific transcription factors	Enhanced drought tolerance and reduced stomatal conductance	Mutants showed drought-tolerant phenotype under the 10-day watering cessation treatment.	[56]
Tomato	*SlARF4*	Auxin response factors	Enhanced drought tolerance and stem thickness	Mutants showed drought-tolerant phenotype under the 12-day watering-off treatment.	[57]
Arabidopsis	*AtAITR* family	ABA-induced transcription repressors	Enhanced drought and salt tolerance, reduced ABA sensitivity	Mutants showed drought-tolerant phenotype after 12-day watering off treatment and 2 days of rewatering.	[58]
Arabidopsis	*AtOST2*	Stomatal opening regulator	Enhanced drought tolerance and stomatal closure	Mutants showed a lower water loss rate than the wild type after 5 h of normal treatment.	[59]
Arabidopsis	*AREB1*	ABA-responsive element-binding protein	Enhanced drought tolerance and chlorophyll content	Mutants showed drought-tolerant phenotype under 20% humidity treatment or 20-day cessation of watering.	[60]
Maize	*ARGOS8*	Negative regulator of ethylene responses	Enhanced drought tolerance, increased grain yield	Mutants sown on soil with only normal 15% moisture showed drought-tolerant phenotype.	[61]
Rice	*OsERA1*	ABA signaling and the dehydration response	Enhanced response to drought stress through stomatal regulation	Mutants showed drought-tolerant phenotype under the 8-day watering-off treatment.	[62]
Rice	*OsSRL1,2*	Regulation of leaf rolling	Enhanced drought tolerance and ABA level	Mutants showed drought-tolerant phenotype under 30-day water-deficient treatment.	[63]
Tomato	*SlNPR1*	A special receptor of salicylic acid	Reduced drought tolerance, increased stomatal aperture	Mutants showed drought-sensitive phenotype without watering for 6 consecutive days.	[64]
Tomato	*SlMPK3*	Mitogen-activated protein kinases	Reduced drought tolerance, severe wilting symptom	Mutants showed drought-sensitive phenotype without watering for 5 consecutive days.	[65]
Soybean	*GmMYB118*	MYB transcription factor family	Reduced drought and salinity tolerance	Mutants showed drought-sensitive phenotype after 14-day no water treatment.	[66]
Rice	*OsPUB67*	U-box E3 ubiquitin ligase	Reduced drought tolerance	Mutants showed drought-sensitive phenotype after 10-day no water treatment at tillering stage.	[67]
Rice	*OsSAPK2*	Osmotic stress/ABA–activated protein kinase	Reduced drought tolerance, ROS scavenging was inhibited	Mutants showed drought-sensitive phenotype after 7-day no water treatment.	[68]
Rice	*OsRR22*	Involved in both cytokinin signal transduction and metabolism	Enhanced salinity tolerance	Mutants showed salinity-tolerant phenotype under concentrations of 0.75% NaCl solution treatment.	[69]
Rice	*OsVDE*	Key enzyme of xanthophyll cycle	Enhanced salinity tolerance, reduced water loss	Mutants showed salinity-tolerant phenotype at 100 mM NaCl application.	[70]
Rice	*OsDST*	Drought and salt tolerance gene	Enhanced salinity tolerance, showed significantly broader leaf width and enhanced leaf area	Mutants showed salinity-tolerant phenotype at 200 mM NaCl application.	[71]
Rice	*OsNAC041*	NAC transcription factor	Reduced salinity tolerance, enhanced MDA content	Mutants showed salinity-sensitive phenotype at 150 mM NaCl application.	[72]
Tomato	*SlHyPRP1*	A subgroup of putative plant cell wall glycoproteins	Enhanced salinity tolerance and stem length	Mutants showed salinity-tolerant phenotype at 100 mM and150 mM NaCl application.	[73]
Tomato	*SlARF4*	Auxin response factor	Enhanced salinity tolerance, delayed flowering, increased height and leaf curling	Mutants showed salinity-tolerant phenotype at 250 mM NaCl application.	[74]
Arabidopsis	*AtAITR*	ABA-induced transcription repressors	Enhanced salinity tolerance, reduced ABA sensitivity	Mutants showed salinity-tolerant phenotype at 150 mM NaCl application.	[58]
Arabidopsis	*ACQOS*	A toll-interleukin1 receptor-nucleotide-binding leucine-rich repeat class protein	Enhanced salinity tolerance and chlorophyll content	Mutants showed salinity-tolerant phenotype at 250 mM NaCl application.	[75]
wheat	*TaHAG1*	Histone acetyltransferase	Reduced salinity tolerance, more chlorotic leaves and higher Na^+^ content in the mutants	Mutants showed salinity-sensitive phenotype at 200 mM NaCl application.	[76]
Potato	*Coilin*	A main structural protein controlling the formation, composition, and activity of subnuclear Cajal bodies	Enhanced salinity tolerance, slower yellowing and leaf fall	Mutants showed salinity-tolerant phenotype at 300 mM NaCl application.	[77]
Soybean	*GmAITR*	ABA-induced transcription repressors	Enhanced salinity tolerance, more sensitivity to ABA	Mutants showed salinity-tolerant phenotype at 200 mM NaCl application.	[78]

**Table 2:** Comparison of the delivery of the CRISPR/Cas9 expression cassette.

Delivery Method	Characteristics	Limitations
*Agrobacterium*-based tissue culture method	*Agrobacterium* infects plant cells and delivers DNA, conventional	Time-consuming and laborious, regeneration protocols required
Viral delivery method	Use of virus-based vectors for transient expression	Limited loading capacity, species-specific restriction
Nanoparticle delivery method	Use of nanoparticle–DNA complex for delivery of DNA	Difficult to deliver the complete CRISPR/Cas9 expression cassette through the cell wall
*In planta* particle bombardment method	DNA-, RNA-, and/or protein-coated particles bombarding plant tissue	Regeneration required

## Data Availability

Not applicable.
